# Clinical Significance of Altered Expression of β-Catenin and E-Cadherin in Oral Dysplasia and Cancer: Potential Link with ALCAM Expression

**DOI:** 10.1371/journal.pone.0067361

**Published:** 2013-06-28

**Authors:** Jatinder Kaur, Meenakshi Sawhney, Siddhartha DattaGupta, Nootan Kumar Shukla, Anurag Srivastava, Paul G. Walfish, Ranju Ralhan

**Affiliations:** 1 Alex and Simona Shnaider Laboratory in Molecular Oncology, Department of Pathology and Laboratory Medicine, Samuel Lunenfeld Research Institute, Mount Sinai Hospital, Toronto, Ontario, Canada; 2 Department of Biochemistry, All India Institute for Medical Sciences, Ansari Nagar, New Delhi, India; 3 Department of Pathology, All India Institute for Medical Sciences, Ansari Nagar, New Delhi, India; 4 Department of Surgical Oncology, Institute of Rotary Cancer Hospital, All India Institute for Medical Sciences, Ansari Nagar, New Delhi, India; 5 Department of Surgical Disciplines, All India Institute for Medical Sciences, Ansari Nagar, New Delhi, India; 6 Joseph and Mildred Sonshine Family Centre for Head and Neck Diseases, Department of Otolaryngology – Head and Neck Surgery, Mount Sinai Hospital, Toronto, Ontario, Canada; 7 Department of Pathology and Laboratory Medicine, Mount Sinai Hospital, Toronto, Ontario, Canada; 8 Department of Medicine, Endocrine Division, Mount Sinai Hospital, Toronto, Ontario, Canada; 9 Department of Otolaryngology-Head and Neck Surgery, University of Toronto, Toronto, ON, Canada; Sapporo Medical University, Japan

## Abstract

**Background:**

Perturbations in cell adhesion molecules are linked to alterations in cadherin-catenin complexes and likely play major roles in invasion and metastasis; their impact on early precancerous stages remains yet unknown. We showed ALCAM overexpression in early oral lesions and its cytoplasmic accumulation in oral squamous cell carcinoma (OSCC) to be a predictor of disease progression and poor prognosis. This study tested the hypothesis that alterations in E-cadherin and β -catenin expressions are early events in oral tumorigenesis, associated with disease prognosis, and correlate with perturbations in ALCAM expression.

**Methods:**

Expressions of E-cadherin and β-catenin were analyzed in the same cohort of 105 OSCCs, 76 oral lesions and 30 normal oral tissues by immunohistochemistry and correlated with clinicopathological parameters and prognosis. The effect of siRNA mediated ALCAM knockdown on E-cadherin and β -catenin was determined using western blot, confocal microscopy and RT-PCR analysis in oral cancer cells.

**Results:**

Significant loss of membranous E-cadherin and β-catenin expression was observed from normal, hyperplasia, dysplasia to OSCCs (p_trend_ <0.001); and correlated with cytoplasmic ALCAM accumulation in OSCCs (p  = 0.006). Multivariate analysis revealed β-catenin membrane loss and ALCAM/β-catenin_nuclear/cytoplasmic_ accumulation to be significant predictors for late clinical stage (p<0.001, OR = 8.7; p = 0.006, OR = 9.9, respectively) and nodal metastasis (p = 0.003, OR = 3.8; p = 0.025, OR = 3.4 respectively). Cox’s regression showed E-cadherin membrane loss/ALCAM cytoplasmic expression [p<0.001; HR = 4.8] to be independent adverse prognosticators in OSCCs. siRNA mediated silencing of ALCAM resulted in concurrent increase in E-cadherin and β-catenin both at the transcript and protein levels.

**Conclusions:**

Losses of E-cadherin and β-catenin expressions are early events in oral tumorigenesis; their associations with aggressive tumor behavior and disease recurrence underscore their potential as prognostic markers. Correlation of loss of E-cadherin and β-catenin with cytoplasmic ALCAM accumulation both *in vitro* and in *in vivo* suggests that these dynamic changes in cell adhesion system may play pivotal role in oral cancer.

## Introduction

Perturbations in orchestrated modulation of cell adhesion cause defects in tissue architecture that play critical roles in cancer development [1]–[3]. E-cadherin is localized on the surface of epithelial cells in regions of cell-cell contact known as adherens junctions and is implicated in cell-cell adhesion in epithelial tissues [4]–[6]. β-catenin interacts with cadherins through its cytoplasmic domain. α-catenin connects the E-cadherin and β-catenin complex to actin filaments. The dissociation of E-cadherin-catenin complex from cell membrane is important in malignant progression. In many epithelial cancers, membranous E-cadherin is lost and β-catenin dissociates in the cytoplasm and accumulates in the nucleus as a transcription factor, concomitantly with tumor progression [5]. Down-regulation of membranous E-cadherin and β-catenin, and cytoplasmic/nuclear accumulation of β-catenin have been previously reported in several cancers and hold promise as prognostic markers [7].

The development of oral squamous cell carcinoma (OSCC) is a multistep process involving interactions between several factors such as tobacco-associated intra-oral carcinogens, areca nut, betel quid and alcohol consumption, and/or viral infections [8], [9]. OSCCs are often preceded by clinically evident lesions often leukoplakia, and the risk of multiple cancers is 5–10 times greater in patients with OSCCs preceded by leukoplakia [8], [10]. These lesions often progress to cancer if untreated [11]. An average annual transformation rate of 1% has been proposed based on several studies reporting 5% transformation observed in 5 years [11], [12]. In India, over 80% of OSCCs arise from existing oral lesions (OLs) [13], [14]. The ability to predict outcome of OLs remains a major challenge for early intervention. Early detection of OLs that will develop into invasive tumors is necessary to improve the poor prognosis of oral cancer patients.

Dynamic changes in cell adhesion manifested by dissociation of membranous E-cadherin-β-catenin complex are implicated in loss of epithelial cohesion as an important event in invasion and metastasis. Activated leukocyte cell adhesion molecule (ALCAM)/MEMD/CD166) is a transmembrane glycoprotein of immunoglobulin superfamily that mediates cell-cell adhesion through both homophilic (ALCAM-ALCAM) and heterophilic (ALCAM-CD6) interactions [15], [16]. We demonstrated ALCAM expression is increased in OLs and its cytoplasmic accumulation in OSCC is a predictor of disease progression and poor prognosis [17]. Herein, we sought to investigate the clinical significance of E-cadherin and β-catenin expression in serial tissue sections of the same set of patients from different disease stages by immunohistochemistry, to identify stage-specific protein alterations and determined their relationship with ALCAM expression. The experimental evidences in support of correlations between perturbations in ALCAM expression and alterations in E-cadherin and β-catenin expressions were provided by short interfering RNA (siRNA) mediated ALCAM knockdown and determining the change in expression of all these three proteins both at the transcript and protein levels.

## Materials and Methods

### Tissue Specimens

This study was approved by Human Ethics Committee of All India Institute of Medical Sciences, New Delhi, India. For immunohistochemical analysis surgically resected tissues or biopsy specimens from OSCC, dysplasia, hyperplasia and histologically normal oral tissues were obtained from Surgical Oncology Unit of Dr. B.R. Ambedkar Institute Rotary Cancer Hospital, All India Institute of Medical Sciences, New Delhi, India, with prior informed written consent of the patients.

### Clinicopathological Characteristics of Patients

One hundred and five primary OSCC patients (age range, 29–75 years, mean age 40 years) undergoing curative oral cancer surgery, at the Surgical Oncology Unit of Dr. B.R. Ambedkar Institute Rotary Cancer Hospital, All India Institute of Medical Sciences, New Delhi, India were enrolled in this study after obtaining prior written consent of the patients. The clinical and pathological data including clinical TNM staging (tumor, node, metastasis based on Union International Center le Cancer TNM classification), site of the lesion, histopathological differentiation, age, and gender were recorded in a pre-designed performa as described previously [17]. The diagnosis was based on clinical examination and histopathological analysis of the tissue specimens. The site distribution of OSCC cases was: buccal mucosa (36), tongue (35), alveolus (12), lip (6) and other sites (16) including ginigivobuccal sulcus, hard palate, soft palate, retromolar trigone and floor of the mouth. The tumors were histologically graded as well, moderately or poorly differentiated SCCs. Biopsies from OLs with histological evidence of hyperplasia (56 cases) and dysplasia (20 cases) were also included in this study. The site distribution of OLs was: buccal mucosa (53), tongue (12), alveolus (5), lip (4) and ginigivobuccal sulcus (2). Thirty non-malignant tissues taken from a distant site of OSCCs (with histologically confirmed normal oral epithelium hither to referred to as oral normal tissues) were also evaluated for ALCAM expression. After excision, tissues were immediately snap frozen in liquid N_2_ and stored at −80°C till further use and one piece was collected in 10% formalin and embedded in paraffin for histopathological and immunohistochemical analyses.

### Immunohistochemistry

Paraffin embedded sections (5 µm thickness) of human oral tissue specimens were stained with hematoxylin and eosin for histopathological analysis, and immunostaining was done on serial sections as described previously by Sawhney *et al*. [17]. Briefly, tissue sections after antigen retrieval in citrate buffer were incubated with anti-E-cadherin or anti-β-catenin antibodies (0.2 µg/ml) (Santa Cruz Biotechnology Inc., Santacruz, CA) for 16 h at 4°C. Antibodies were detected using biotinylated secondary antibody and peroxidase labeled Streptavidin complex using Dako LSAB plus kit (Dako Labs, Glostrup, Denmark) using diaminobenzidine (DAB) as chromogen. In negative controls, the primary antibody was replaced by non-immune IgG of the same isotype to ensure specificity. Breast cancer tissue sections with known immunopositivity for E-cadherin or β-catenin were used as positive controls in each batch of sections analyzed.

### Positive Criterion for Immunohistochemical Staining

The immunostaining was evaluated in randomly selected five non-overlapping areas of the tissue sections with more than 80% epithelial cells. For E-cadherin, specific staining in the membrane was defined as positive staining. The slides were scored as follows: ≥50% tumor cells showing immunoreactivity were graded as positive for E-cadherin expression, while those showing immunostaining in <50% tumor cells were graded as negative [18]. For β-catenin protein expression, specific staining in the membrane/cytoplasm/nuclei was defined as positive staining. For membrane staining the slides were scored as follows: ≥50% tumor cells showing immunoreactivity were graded as positive, while those showing immunostaining in <50% tumor cells were graded as negative [18]. For cytoplasmic/nuclear staining of β-catenin the slides were scored as follows: 0, <10% tumor cells showing immunoreactivity; 1 = 10–30% tumor cells showing immunoreactivity; 2, ≥31–50% tumor cells showing immunoreactivity; 3, >50% tumor cells showing immunoreactivity. The immunohistochemical investigation was blind, i.e. the slides were coded and pathologist did not have prior knowledge of the local tumor burden, lymphonodular spread and grading of the OSCCs while scoring the immunoreactivity.

### Follow-up Study

Seventy-two of the 105 OSCC patients, who underwent treatment of primary OSCC from 2002–2005, could be followed regularly in the follow up clinic, while 33 patients were lost to follow up. Survival status of patients was verified and regularly updated from Tumor Registry records, Institute of Rotary Cancer Hospital, as of December 2010. As per our protocol, OSCC patients with T_1_ and T_2_ tumors were treated with radical radiotherapy or surgery alone, whereas majority of patients with T_3_ and T_4_ disease were treated using a combination of radical surgery followed by postoperative radical radiotherapy as described [19]. The patients were followed up periodically and time to recurrence was recorded. If a patient died during the follow-up, patient survival time was censored at the time of death. Medical history, clinical examination, and radiological evaluation were used to determine whether death resulted from recurrent cancer (relapsing patients) or from any other cause. Disease-free survivors were defined as patients free from clinical and radiological evidence of local, regional, or distant relapse at the time of the last follow-up. Loco-regional relapse/death was observed in 32/72 (44%) patients monitored in this study. Patients who did not show recurrence were alive till the end of the follow up period. Among the 33 patients that were lost to follow up, the number of deaths could not be ascertained; therefore overall survival could not be considered as a separate parameter in our study. Only disease free survival of the patients was studied. Disease free survival was expressed as the number of months from the date of surgery to the loco-regional relapse. Patients were monitored for a period of median 24 months and maximum 91 months.

### Statistical Analysis

The immunohistochemical data were subjected to statistical analysis using SPSS 17.0 software (Chicago IL). The relationships between E-cadherin, β-catenin and ALCAM expression and clinicopathological parameters was tested in univariate analysis by Chi-Square test, Chi-Square test for trend, Fisher’s exact test and logistic regression analysis. To determine independent predictors for tumorigenesis, logistic regression analysis was carried out in stepwise manner for the individual variables, clinicopatholgical parameters and the proteins E-cadherin, β-catenin and ALCAM. Different combinations of variables were generated to assess the association between these combinations and patient prognosis. Follow-up studies were analyzed by Kaplan-Meier, and Cox’s Proportional Hazards test. Only disease free survival was evaluated in the present study, as the number of deaths due to disease progression did not allow a reliable statistical analysis. The association between patient outcome and variables was assessed by log–rank test. Two sided p values were calculated and p<0.05 was considered to be significant.

### Short Interfering RNA–mediated ALCAM Knockdown

Human head and neck squamous carcinoma cell line, SCC-4 cells were maintained in DMEM containing 10% FBS, 100 µg/ml streptomycin, 100U/ml penicillin at 37°C in humidified atmosphere of 5% CO_2_. SCC-4 cells were transiently transfected using Hiperfect reagent (Qiagen) and a blunt-ended duplex of the RNA oligonucleotides, 5′- AAG CCC GAU GGC UCC CCA GUA UU-3′and 5′-AAU ACU GGG GAG CCA UCG GGC UU-3′. SCC-4 cells were treated with varying concentrations of siRNA (1–15 nM) and with varying concentrations of Hiperfect reagent (1–4.5 µL). ALCAM siRNA (15 nM) with 4.5 µL of Hiperfect reagent for 48 h was found to be the best concentration with maximum transfection efficiency (95%) and maximum silencing of ALCAM gene expression (data not shown). Forty-eight hours post-transfection, siRNA-treated cells were used in subsequent experiments.

### Reverse Transcription-PCR Analysis

Total RNA was isolated from SCC-4 cells (control and siRNA transfectants) as described earlier [20]. PCR amplification was carried out in a total volume of 20 µl containing 3 µl reverse transcribed cDNA, 10X PCR buffer, 10 mM dNTPs, 20 µM of each primer and 1unit of Taq polymerase, (Gibco BRL, Gaithersburg, MD). After 5 minutes of initial denaturation, 35 amplification cycles of 1 minute at 94°C, 2 minutes at specific annealing temperature and 1 minute at 72°C were carried out, followed by a 10 minutes elongation at 72°C. For amplification of ALCAM cDNA forward primer, 5′-GTC TGG GCA ATA GTG ACT CC-3′ and reverse primer 5′-AAC CAT TGC AAG TGG AAA CC-3′ were used. For E-cadherin cDNA, forward primer 5′-CAG CAC GTA CAC AGC CCT AA-3′ and reverse primer 5′-ACC TGA GGC TTT GGA TTC CT-3′ were used. For β-catenin cDNA, forward primer 5′-GAA ACG GCT TTC AGT TGA GC-3′ and reverse primer 5′-CTG GCC ATA TCC ACC AGA GT -3′ were used. β-actin was used as an internal control to ensure that equal amount of RNA was used in control and siRNA transfected cells. For β-actin cDNA, forward primer 5′- CAG CCA TGT ACG TTG CTA TCC AG -3′ and reverse primer 5′- GTT TCG TGG ATG CCA CAG GAC -3′ were used. PCR products were separated on 1.5% agarose gel, stained with ethidium bromide, and visualised with AlphaEase FC Software (version 3.1.1) The intensities of PCR bands were quantified with Chemi Imager IS-4400 (Alpha Innotech Corp, CA) as described earlier [14].

### Immunoblotting

ALCAM siRNA transfected SCC-4 cells for 48 h and untreated control cells were used for the preparation of cell extracts by boiling the cell pellet in SDS-lysis buffer. Briefly, whole cell extract (100 µg protein/lane) were resolved by SDS-PAGE and transferred onto nitrocellulose membrane [21]. The nitrocellulose membranes were blocked with 10% non-fat milk overnight at 4°C and probed with anti-ALCAM; E-cadherin and β-catenin antibodies (Santa Cruz Biotechnology Inc., Santacruz, CA) for 2 h at 37°C. Thereafter, the membranes were probed with secondary antibodies to these proteins (anti-goat 1∶5000 and anti-mouse 1∶2000 dilution). The blots were reprobed with α-tubulin to normalize for equal protein loading. The blots were developed using Enhanced chemiluminescence Reagent (ECL) (Amersham, Buckinghamshire, UK) as described [21].

### Confocal Laser Microscopy

SCC-4 cells grown on coverslips were treated with ALCAM siRNA for 48 h and processed for confocal laser microscopy as described previously [21]. Cells were rinsed in Dulbecco’s PBS (DPBS), fixed in methanol for 5 min. at −20°C and incubated with anti-ALCAM, E-cadherin and β-catenin antibodies (10 ng/ml) obtained from SantaCruz Biotechnologies Inc., Santacruz, CA. After rinsing in DPBS, the coverslips were incubated with biotinylated secondary antibody (anti-rabbit/goat, LSAB+ Kit, DAKO Cytomations, Glostrup, Denmark) for 45 min. at 37°C followed by incubation with streptavidin-conjugated fluorochrome, fluorescein isothiocyanate (FITC) (DAKO Cytomations, Denmark). Thereafter, the coverslips were counterstained with propidium iodide (PI) (10 mg/ml; Sigma-Aldrich, MO) for 30 sec. Coverslips were then rinsed and mounted in mounting medium. Slides were examined with Confocal Laser Scanning Microscope as described previously [21].

## Results

### Immunohistochemical Analysis of E-cadherin Membranous Expression in Normal Oral Tissues, Hyperplasia, Dysplasia and OSCC

**Figure 1:**
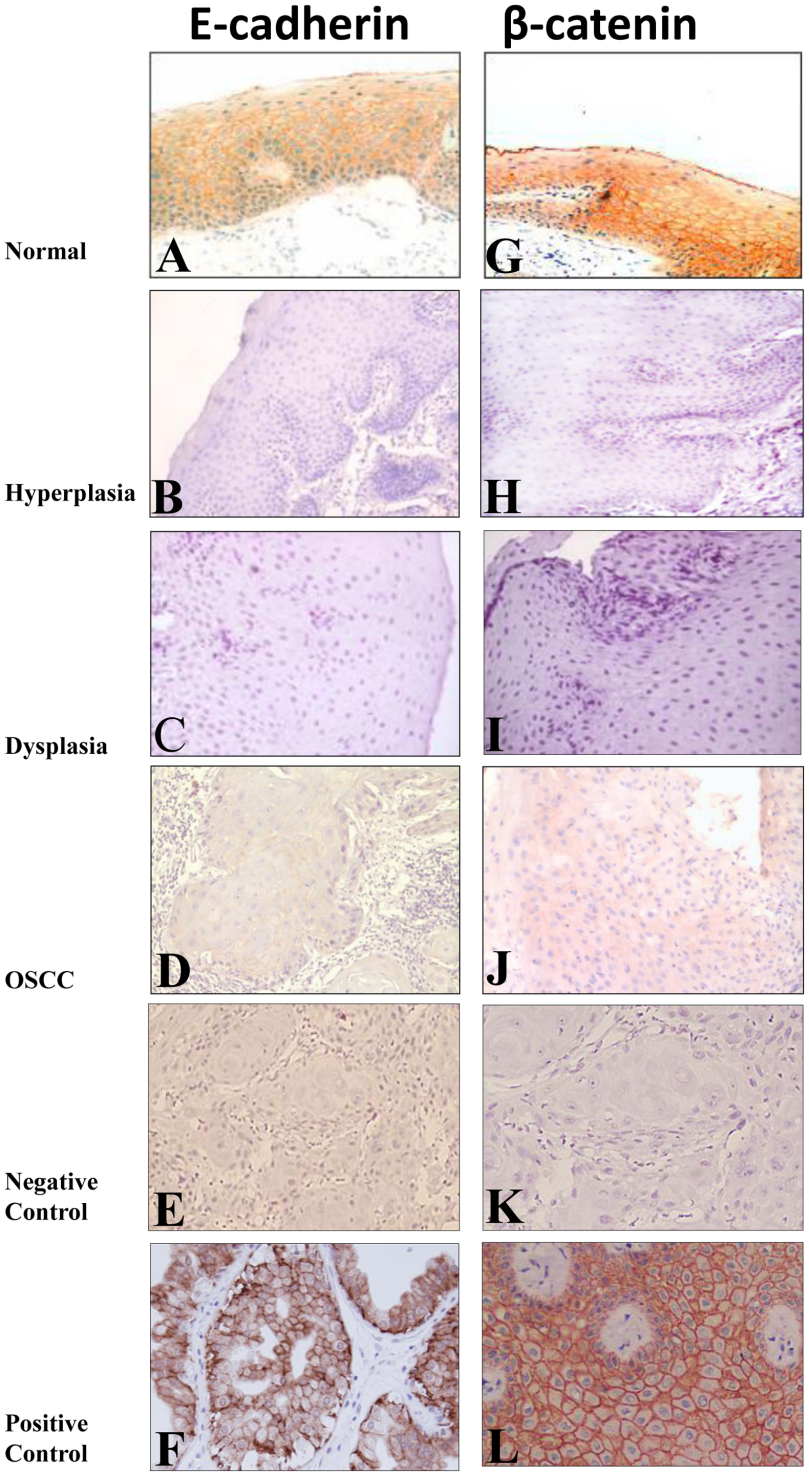
Representative oral tissue sections immunostained for E-cadherin and β-catenin. Histologically normal tissue sections showing membranous immunoreactivity for E-cadherin (A), and β-catenin (G) proteins. Hyperplastic (B), dysplastic (C), OSCC (D) tissue sections depicting loss of membranous staining for E-cadherin. Hyperplastic (H), dysplastic (I), OSCC (J) tissue sections depicting loss of membranous staining for β-catenin. In negative controls, for E-cadherin (E) and β-catenin (F), the primary antibody was replaced by non-immune IgG of the same isotype to ensure specificity. Breast cancer tissue sections used as positive control showed membrane staining for E-cadherin (K) and β-catenin (L) proteins. Original magnification X 200.

E-cadherin expression in the membrane of epithelial cells was observed in 27/30 (90%) of the histologically normal oral tissues with occasional cytoplasmic expression (Table 1, Figure 1A). Significant loss of E-cadherin membranous expression was observed in hyperplasia [19/56, 34% (Figure 1B) as compared to the normal oral tissues (3/30, 10%), [p = 0.015; OR = 4.6, 95% C.I. = 1.2–17.2] and in 12/20, 60% dysplasia (Figure 1C) [H/D: p = 0.06; OR = 2.9, 95% C.I. = 1.0–8.4]. Chi-Square trend analysis showed significant loss of E-cadherin membranous expression in tissues from different stages of oral cancer development (normal, hyperplasia, dysplasia and invasive cancer; p_trend_ <0.001). Loss of E-cadherin membranous expression was observed in 61/105 (58%) OSCCs (Table 1, Figure 1D) and was associated with late clinical stage (p = 0.006, OR = 3.1, 95% C.I = 1.3–7.0), increased tumor burden (p = 0.03; OR = 2.4, 95% C.I. = 1.1–5.3), and nodal metastasis (p = 0.04; OR = 2.4, 95% C.I. = 1.1–5.3).

**Table 1:** Analysis of E-cadherin and β-catenin expression in normal oral tissues, hyperplasia, dysplasia and OSCCs: Correlation with clinicopathological parameters.

Clinicopathological Features	Total Cases. N	E-cadherin Membrane loss n (%)		p_value_ OR	β-catenin membrane loss n (%)		p_value_ OR	β-catenin cytoplasmic/nuclear positivity n (%)		p_value_ OR
**Normal (N)**	30	3	(10)		3	(10)		–		
**Hyperplasia**	56	**19**	**(34)**	**0.015 4.6**	**20**	**(36)**	**0.01 5.0**	**10**	**(18)**	
**Dysplasia**	20	12	(60)	**0.06 2.9**	14	(70)	**0.008 4.2**	8	(40)	**0.046 3.1**
**OSCCs**	**105**	**61**	**(58)**		**65**	**(62)**		**28**	**(27)**	
**Differentiation**										
WDSCC	61	37	(61)		41	(67)		16	(26)	
MDSCC+PDSCC	44	24	(55)		24	(55)		12	(27)	
**Tumor Stage** a										
T_1_+ T_2_	49	**23**	**(47)**		**22**	**(45)**		**8**	**(16)**	
T_3_+ T_4_	56	38	(68)	**0.03 2.4**	43	(77)	**0.001 4.1**	20	(36)	**0.025 2.8**
**Nodal Status**										
N_0_	59	**29**	**(49)**		**29**	**(49)**		12	(20)	
N_1_	46	32	(70)	**0.04 2.4**	36	(78)	**0.003 3.7**	16	(35)	
**Clinical Stage**										
I+II	39	**16**	**(41)**		**13**	**(33)**		**5**	**(13)**	
III+IVa	66	45	(68)	**0.006 3.1**	52	(79)	**0.001 7.4**	23	(35)	**0.014 3.6**

a TNM stage. OR, Odd’s ratio.

### Association of Loss of Membranous E-cadherin Expression with Disease Outcome

**Figure 2:**
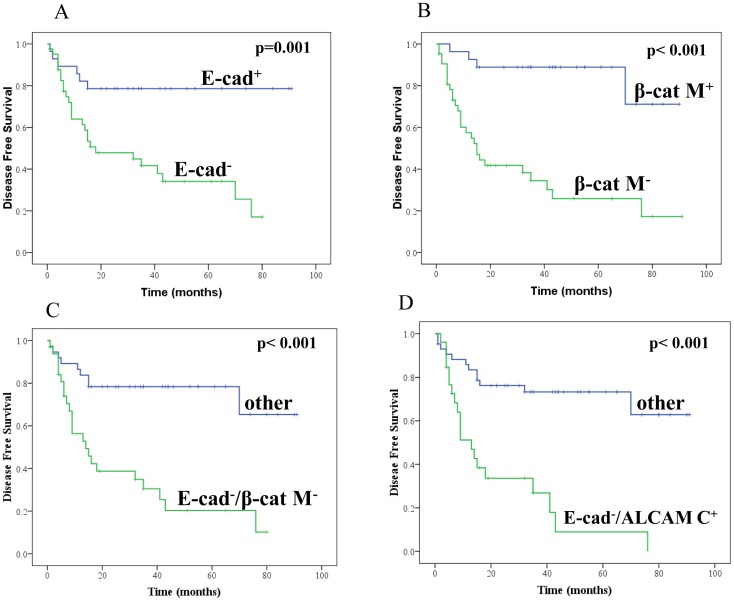
Kaplan–Meier estimation of cumulative proportion of no tumor recurrence. **A:** loss of E-cadherin membranous (E-cad^-^) expression, **B:** Loss of β-catenin membranous (β-catM^-^) expression, **C:** loss of E-cadherin and β-catenin membranous (E-cad^−/^β-cat M^-^) expression, **D:** ALCAM cytoplasmic positive and E-cadherin membrane loss (E-cad^−/^ALCAM C^+^).

Seventy-two OSCC patients could be followed up for a maximum period of 91 months (median 24 months). Kaplan–Meier survival analysis and Cox’s proportional hazard model revealed that patients with E-cadherin positive tumors had increased median disease-free survival (DFS) of 74 months as compared to those with loss of membranous immunopositivity (median DFS = 18 months) [p = 0.001; HR = 3.9; 95% CI = 1.6–9.6] (Figure 2A).

### Immunohistochemical Analysis of β-catenin Expression in Normal Oral Tissues, Hyperplasia, Dysplasia and OSCC

β-catenin was mainly present in the membrane of epithelial cells in 27/30 (90%) of histologically normal oral tissues; occasionally cytoplasmic/nuclear staining was also observed (Table 1, Figure 1G). Significant loss of β-catenin membrane expression was observed in hyperplasia [20/56, 36%, p = 0.01; OR = 5.0, 95% C.I. = 1.3–18.6, (Figure 1H)] as compared to normal oral tissues (3/30, 10%), and in dysplasia as compared to hyperplasia [14/20, 70%, (Figure 1I), p = 0.008; OR = 4.2, 95% C.I. = 1.4–12.6]. Significant increase in cytoplasmic/nuclear accumulation of β-catenin was observed in dysplasia as compared to hyperplasia (p = 0.046; OR = 3.1; 95% C.I. = 1.0–9.5) (Table 1). Chi-Square trend analysis showed significant loss of β-catenin membranous expression and increase in its cytoplasmic/nuclear accumulation in tissues from different stages of oral cancer (normal, hyperplasia, dysplasia, and invasive cancers; p_trend_ <0.001 and p_trend = _0.003 respectively).

Loss of β-catenin membranous expression was observed in 65 of 105 (62%) OSCCs (Figure 1J, Table 1) and was associated with late clinical stage (p = 0.001, OR = 7.4, 95% C.I. = 3.1–18.1), increased tumor burden (p = 0.001, OR = 4.1, 95% C.I. = 1.8–9.4) and nodal metastasis (p = 0.003, OR = 3.7, 95% C.I. = 1.6–8.9). In OSCCs 28 of 105 (27%) cases showed cytoplasmic/nuclear accumulation of β-catenin and was associated with late clinical stage (p = 0.014, OR = 3.6, 95% C.I. = 1.2–10.6) and tumor burden (p = 0.025, OR = 2.8, 95% C.I. = 1.1–7.2).

### Association of Loss of Membranous β-catenin Expression with Disease Outcome

Loss of membranous β-catenin immunopositivity was associated with reduced disease free-survival (median DFS = 15 months) in comparison with patients with β-catenin membrane positive tumors (median DFS = 78 months) [p<0.001; HR = 7.8; 95% CI = 2.7–22.3] (Figure 2B).

### Association between E-cadherin and β-catenin in Oral Lesions and OSCCs

Significant association was observed between loss of membranous E-cadherin and β-catenin expressions in OLs (p = 0.001, OR = 16.7, 95% C.I. = 5.3–52.7, Table 2) and OSCCs (p = 0.001, OR = 8.8, 95% C.I. = 3.6–21.7, Table 2). Loss of membranous E-cadherin and β-catenin when taken as one phenotype (E-cad^−/^β-cat M^-^) correlated significantly with advanced tumor stage (p = 0.004, OR = 3.2, 95% C.I. = 1.4–7.1), nodal metastasis (p = 0.005, OR = 3.1, 95% C.I. = 1.4–6.9) and late clinical stage (p<0.001, OR = 5.5, 95% C.I. = 2.2–13.4). Chi-Square trend analysis showed significant loss of membranous E-cadherin and β-catenin (E-cad^−/^β-cat M^-^) in different stages of oral carcinogenesis (normal, hyperplasia, dysplasia and invasive cancer; p_trend = _0.008). Furthermore, loss of E-cad^−/^β-cat M^-^ immunopositivity was significantly associated with reduced disease free-survival [median DFS = 14 months as compared to β-catenin and E-cadherin membrane positive tumors with median disease-free survival of 71 months (p<0.001; HR = 4.7; 95% CI = 2.1–10.1)] (Figure 2C).

**Table 2:** Associations among E-cadherin, β-catenin and ALCAM expression in oral lesions and OSCCs.

Oral Lesions	p_value_	OR	95% C.I.
E-cad^−/^β-cat M^-^	0.001	16.7	5.3–52.7
β-cat M^−/^ALCAM^+^	0.018	3.3	1.2–8.8
β-cat M^−/^ALCAM C^+^	0.008	3.6	1.4–9.3
**OSCCs**			
E-cad^−/^β-cat M^-^	0.001	8.8	3.6–21.7
E-cad^−/^ALCAM C^+^	0.038	2.2	1.0–5.0
β-cat M^−/^ALCAM^+^	0.001	3.9	1.7–9.0
β-cat M^−/^ALCAM C^+^	0.002	3.7	1.6–8.7
E-cad^-^β-cat M^−/^ALCAM^+^	0.002	3.6	1.6–8.4
E-cad^-^β-cat M^−/^ALCAM C^+^	0.006	3.2	1.4–7.1

### Association between E-cadherin, β-catenin and ALCAM Expression in Oral Lesions

To explore the significance of ALCAM in biological development of invasive cancer and metastasis, it is important to determine its correlation with perturbations in expression of adherens junction components. Associations among protein expressions of E-cadherin, β-catenin and ALCAM were determined (Table 2) as ALCAM protein expression had also been analyzed in the same cohort of patients in an earlier study [17]. Loss of β-catenin membranous expression was found to be significantly associated with overall (p = 0.018; OR = 3.3, 95% C.I. = 1.2–8.8, Table 2) and cytoplasmic (p = 0.008; OR = 3.6, 95% C.I. = 1.4–9.3, Table 2) ALCAM expression.

Among the hyperplasias analyzed (n = 56) in this study, loss of membranous E-cadherin was found to be significantly associated with loss of β-catenin expression (p<0.001, OR = 24.0, 95% C.I. = 5.6–102). Loss of β-catenin membranous expression was found to be significantly associated with overall ALCAM expression (p = 0.017; OR = 4.5, 95% C.I. = 1.2–16.0). Loss of membrane E-cadherin and β-catenin taken together as one phenotype (E-cad^−/^β-catM^-^) correlated significantly with overall ALCAM immunopositivity in hyperplasia. Among the dysplasias analyzed (n = 20), loss of β-catenin membranous expression was found to be significantly associated with ALCAM cytoplasmic immunopositivity (p = 0.05; OR = 9.0, 95% C.I. = 1.0–100).

### Association between E-cadherin, β-catenin and ALCAM Expression in OSCC

Associations among ALCAM, E-cadherin and β-catenin expressions were determined in OSCC (Table 2). Loss of E-cadherin membranous expression was found to be significantly associated with cytoplasmic ALCAM expression (p = 0.038, OR = 2.2, 95% C.I = 1.0–5.0). Loss of β-catenin membrane expression significantly correlated with overall ALCAM expression (p = 0.001; OR = 3.9, 95% C.I. = 1.7–9.0), and cytoplasmic ALCAM immunopositivity (p = 0.002; OR = 3.7, 95% C.I. = 1.6–8.7). Loss of membrane E-cadherin and β-catenin when taken as one phenotype (E-cad^−/^β-cat M^-^) correlated significantly with overall and cytoplasmic ALCAM immunopositivity in OSCC (Table 2).

### Independent Predictors for Transition from Normal to OL and Cancerous Phenotype

To determine the independent predictors for transition from normal to OL, logistic regression analysis was carried out in a stepwise manner. Of these variables, the parameters that emerged significant in univariate and multivariate analyses are summarized in Table 3. ALCAM cytoplasmic expression (p = 0.024; OR = 11.6; 95% C.I = 1.4–98.2) emerged to be the most significant phenotype for transition of normal oral mucosa to OL. Loss of membranous β-catenin expression emerged as the most significant predictor for transition from hyperplastic to dysplastic lesions (p = 0.01, OR = 4.2, 95% C.I. = 1.3–12.6). Multivariate logistic regression analyses revealed that loss of membranous E-cadherin expression is the most significant phenotype for transition of OL to OSCC (p = 0.02; OR = 2.0; 95%C.I. = 1.1–3.7).

**Table 3:** Association of alterations in ALCAM, E-cadherin and β-catenin expression with evolution of OSCC, clinical parameters and disease prognosis: Multivariate Analysis.

Logistic regression analysis: Transition	p_value_	OR	95% C.I.
**Normal to Oral Lesions**			
ALCAM C^+^	0.024	11.6	1.4–98.2
**Hyperplasia to Dysplasia**			
β-cat M^-^	0.01	4.2	1.3–12.6
**Oral Lesions to Cancer**			
E-cad M^-^	0.02	2.0	1.1–3.7
**Clinical parameters**	**p_value_**	**OR**	**95% C.I.**
**Tumor Stage**			
β-cat M^-^	0.001	4.2	1.8–10.2
β-cat NC	0.027	3.1	1.1–8.3
**Nodal Involvement**			
β-cat M^-^	0.003	3.8	1.6–9.3
ALCAM^+^/β-cat NC	0.025	3.4	1.2–9.7
**Stage**			
β-cat M^-^	0.000	8.7	3.3–22.9
ALCAM^+^/β-cat NC	0.006	9.9	1.9–51.5
**Cox-proportional hazard model for disease free survival analysis**	**p_value_**	**HR**	**95% C.I.**
ALCAM C^+^/E-cad M^-^	0.000	4.8	2.2–9.9

OR, Odd’s ratio; HR, Hazard’s ratio.

### Clinical outcome of patients

#### Independent predictors for clinical parameters, tumor burden, nodal involvement and clinical stage of OSCC

To determine the independent predictors for clinical parameters, tumor burden, nodal involvement and clinical stage, logistic regression analysis was carried out in a stepwise manner for E-cadherin, β-catenin and ALCAM individually, or in combination, in 105 OSCCs **(** Table 3 **).** β-catenin membrane loss (p = 0.001, OR = 4.2; 95% C.I. = 1.8–10.2) and β-catenin nuclear/cytoplasmic accumulation (p = 0.027, OR = 3.1; 95% CI = 1.1–8.3) were the most significant predictors for increased tumor burden. β-catenin membrane loss (p = 0.003, OR = 3.8; 95% C.I. = 1.6–9.3) and overall ALCAM expression/β-catenin nuclear/cytoplasmic accumulation (p = 0.025, OR = 3.4, 95% C.I. = 1.2–9.7) were the most significant predictors for nodal metastasis. Combined markers, ALCAM expression/β-catenin nuclear/cytoplasmic accumulation (p = 0.006, OR = 9.9, 95% C.I. = 1.9–51.5) and β-catenin membrane loss (p<0.001, OR = 8.7; 95% C.I. = 3.3–22.8) were the most significant predictors for late clinical stage (Table 3).

#### Association of alterations in E-cadherin, β-catenin and ALCAM expression with disease outcome

Significant association was observed between reduced disease free-survival and tumor stage [p = 0.03; Hazard Ratio (HR) = 2.5; 95% C.I. = 1.1–5.6].

For possible additive prognostic capacity Kaplan–Meier survival analysis and Cox’s proportional hazard model were used to analyze different combinations of potential biomarkers in order to assess the association of alterations in various biomarkers with clinical outcome. Though several combinations were found to be significant (Table 3), the most significant phenotype that emerged as adverse prognosticator was: increased cytoplasmic ALCAM expression+loss of membranous E-cadherin; ALCAM C^+/^E-cad^−^ (p<0.001; HR = 4.8; 95% CI = 2.2–9.9, Figure 2D) (Table 3).

**Figure 3:**
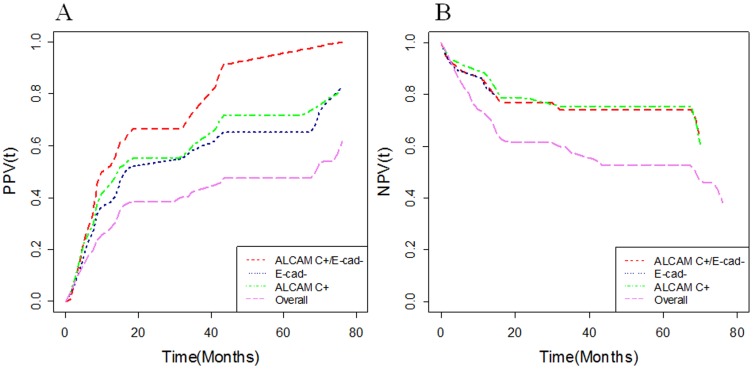
(a) Positive Predictive Values as function of time [PPV(t)] and (b) Negative Predictive Values as function of time [NPV(t)] for 32 OSCC patients with E-cadherin loss of membranous expression (E-cad^−^), ALCAM cytoplasmic (ALCAM C^+^) expression, ALCAM C^+^/E-cad^−^, and for all 72 OSCC patients with survival data (overall) when predicting time to cancer relapse in OSCCs.

Based on our data, the additional prognostic value that ALCAM C^+/^E-cad^−^ provided for predicting cancer recurrence [positive predictive value, (PPV)] or excluding cancer recurrence [negative predictive value, (NPV)] in OSCC patients was measured by the ratios: PPV_relapse_/OSCC (76 months | ALCAM C^+/^E-cad^−^)/PPV_relapse_/OSCC (76 months) = 100/61.6; NPV_relapse_/OSCC (76 months| ALCAM C^+^/E-cad^−^)/NPV_relapse_/OSCC (76 months) = 63.5/46.0 (Figure 3a and 3b). Hence, these findings underscore the potential of ALCAM C^+/^E-cad^−^ as a marker for predicting recurrence in OSCCs.

### siRNA Knockdown of ALCAM and its Effect on E-cadherin and β-catenin

**Figure 4:**
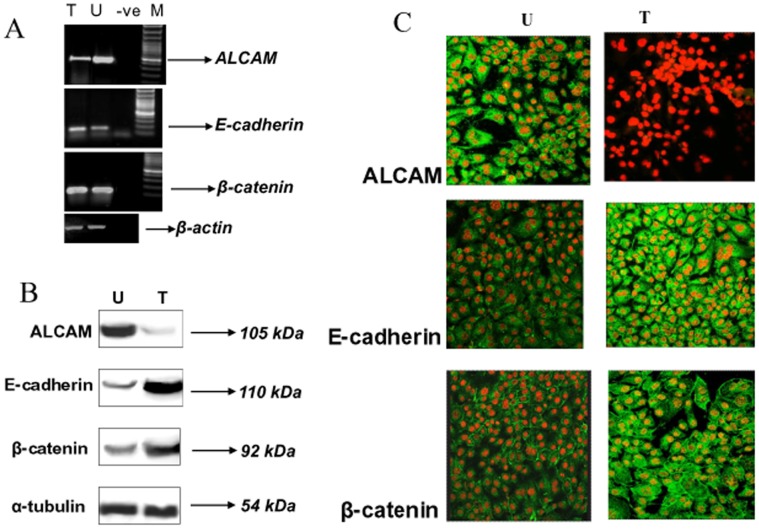
siRNA knockdown of ALCAM and its effect on E-cadherin and β-catenin. **A**: RT-PCR analysis of ALCAM (490bp), E-cadherin (160bp) and β-catenin (167bp) amplicons in siRNA transfected (15 nM, lane T) and control (lane U) SCC-4 cells at 48 h post transfection. β-actin (421bp) transcript was used as control for normalization. **B**: Immunoblot analysis of ALCAM, E-cadherin and β-catenin proteins in SCC-4 cells silenced for ALCAM. SCC-4 cells treated with ALCAM siRNA (15 nM) for 48 h and untreated cells were immunolabelled with respective antibodies and developed using ECL. 7-folds decreased levels of ALCAM (105 kDa) were observed in ALCAM-depleted transfectants (lane T) as compared to untreated SCC-4 cells (lane U); 3-folds increased levels of 110kDa E-cadherin protein (lane T) were observed in ALCAM-depleted transfectants as compared to the untreated SCC-4 cells (lane U); 2-folds increased levels of 92kDa β-catenin protein (lane T) were observed in ALCAM-depleted transfectants as compared to the untreated SCC-4 cells (lane U); α-tubulin (54 kDa) was used as control protein for quantitation. **C**: Expression of ALCAM, E-cadherin, and β-catenin proteins in ALCAM silenced SCC-4 cells. Cells grown on coverslips were treated with ALCAM siRNA, processed for confocal microscopy, immunolabelled with respective antibodies to ALCAM, E-cadherin and β-catenin proteins followed by FITC conjugated secondary antibody (Green fluorescence), nuclei were counterstained with PI (red fluorescence) (Original Magnification X 400).

Significant correlation was observed between ALCAM overexpression and loss of expression of E-cadherin and β-catenin in OLs. These observations suggest that perturbations in ALCAM, E-cadherin and β-catenin expressions in cell adhesion system may play pivotal role in tumor development. To test this hypothesis, head and neck cancer cells (SCC-4) were transiently transfected with siRNA against ALCAM. ALCAM-depleted transfectants (15 nM siRNA) for 48 h showed 3-folds decreased levels of ALCAM transcripts (Figure 4A, lane T) as compared to control cells (Figure 4A, lane U) by RT-PCR analysis. Increased levels of E-cadherin transcripts (1.5 folds) were observed in ALCAM depleted transfectants (Figure 4A, lane T) as compared to control cells (Figure 4A, lane U). Three-fold increase in the levels of β-catenin transcripts were observed in ALCAM depleted transfectants (Figure 4A, lane T) as compared to control cells (Figure 4A, lane U).

ALCAM-depleted transfectants (15 nM siRNA for 48 h) showed 7-folds decreased level of ALCAM protein compared to the untreated control cells as determined by western blot analysis (Figure 4B, lane T). Three-fold increase in the levels of E-cadherin protein was observed in ALCAM depleted transfectants as compared to the control cells. (Figure 4B, lane T). Two-fold increase in the levels of β-catenin protein was observed in ALCAM depleted transfectants as compared to control cells (Figure 4B, lane T).

These results were further corroborated using confocal microscopic analysis; 95% down-regulation was observed in expression of ALCAM protein upon siRNA transfection for 48 h as compared to the control cells (Figure 4C). Increase in the expression of E-cadherin and β-catenin proteins was observed upon siRNA treatment as compared to the control cells (Figure 4C).

**Figure 5:**
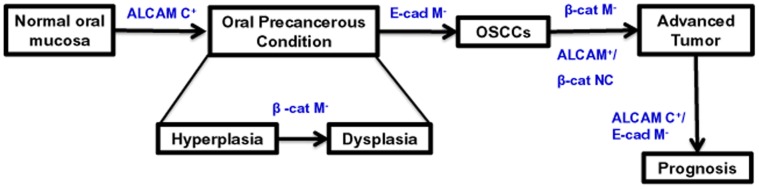
Alterations in expression of ALCAM, E-cadherin and β-catenin proteins in different stages of oral cancer development and prognosis. β-cat NC: β-catenin nuclear/cytoplasmic accumulation, ALCAM M^+^: ALCAM membrane positive, E-cad M^−^: E-cadherin membrane loss.

The results of our study are summarized in figure 5 which depicts the alterations in expression of ALCAM, E-cadherin and β-catenin proteins at different stages in the multistep process of oral tumorigenesis.

## Discussion

The salient findings of the study are:(i) loss of E-cadherin and β-catenin membranous expression, and cytoplasmic/nuclear accumulation of β-catenin are early events in oral tumorigenesis, occurring in pre-neoplastic stages (dysplasia) and sustained in frank malignancy; (ii) loss of E-cadherin/loss of membranous β-catenin (E-cad^−/^β-cat M^−^) correlated significantly with overall ALCAM, and cytoplasmic ALCAM immunopositivity in OSCCs; (iii) a panel of markers, ALCAM overexpression, β-catenin nuclear/cytoplasmic accumulation and β-catenin membrane loss were the most significant predictors for late clinical stage; (iv) Cox regression (multivariate) analysis revealed increased cytoplasmic ALCAM/E-cadherin loss to be the most significant adverse prognosticators for OSCC patients; (v) siRNA mediated ALCAM knockdown resulted in increase in E-cadherin and β-catenin, both at the transcript and protein levels, suggesting the link between these proteins and supporting our clinical findings.

Notably, E-cadherin suppression and aberrant expression of β-catenin have been proposed in the multistep process of oral carcinogenesis in separate studies [22]–[25]. In the metastatic process, reduction in cell-cell adhesion including E-cadherin-catenin cell adhesion complex is an essential step. Kudo *et al*., [26] showed that invasion and metastasis of oral cancer cells require downregulation of E-cadherin and/or degradation of membranous β-catenin. Further, the dynamic but differential epigenetic regulation of E-cadherin is important in the pathogenesis of OSCC [27], [28]. In our study, loss of E-cadherin and β-catenin membrane staining correlated significantly with enhanced tumor invasiveness, late clinical stage, nodal metastasis, and poor prognosis, individually and in combined marker analysis. Down-regulation of E-cadherin and β-catenin accumulation in the cytoplasm/nucleus has been proposed to promote malignant transformation and progression by triggering cyclin D1 expression in breast cancer [29]. Nuclear localization of β-catenin has been shown to be involved in precancerous changes in oral leukoplakia [30]. Wang *et al*., [31] suggested abnormal β-catenin expression in progression of oral carcinomas, lymph node metastasis and cell proliferation in OSCCs. Further, abnormal expression of E-cadherin, β-catenin and alpha-catenin was suggested to be valuable for diagnosis of metastasis in OSCCs [32]. Reduced membranous expression of β-catenin was also found to be an independent marker for lymph node metastasis [33], [34], late clinical stage [35], early local recurrence [36], [37] and poor prognosis in OSCC [22], [38]. Reduced membranous expression of E-cadherin was also found to be an independent marker for poor prognosis in OSCC [25], [33], [34], [38]. Nuclear translocation of β-catenin and decreased expression of E-cadherin has been reported in human papilloma virus-positive tonsillar cancer and proposed to be an early event in human papilloma virus-related tumor progression [39]. The advantage of our study over these studies is the concurrent analysis of E-cadherin, β-catenin and ALCAM in OLs and OSCCs and their correlation with patient outcome.

ALCAM is a progression marker of a variety of cancers [40]–[43], including oral cancer [17] and has emerged as an important diagnostic and prognostic marker. We reported increased expression of ALCAM to be an early event in oral cancer development and its progressive cytoplasmic accumulation correlated with disease progression [17]. Herein we analyzed the expression of well established cell adhesion molecules, E-cadherin and its associated protein β-catenin, in the same cohort and demonstrated a correlation between these proteins and ALCAM protein. Multivariate analysis of expression of these three proteins in clinical specimens from patients diagnosed with oral hyperplasia, dysplasia and SCC, provided the first evidence of concomitant expression of cytoplasmic/reduced membranous expression of ALCAM and loss of membranous E-cadherin and β-catenin in early OLs, dysplasia and sustained in OSCC. Our study showed increased expression of ALCAM in oral hyperplasia, while loss of membranous β-catenin and E-cadherin was significant in dysplasia, suggesting that these molecular alterations are likely to be associated with development of a precancerous state.

Interestingly, loss of membranous E-cadherin/β-catenin and increased cytoplasmic ALCAM expression was significantly associated in OSCCs and correlated with aggressive tumor behavior (enhanced tumor invasiveness, late clinical stage, and nodal metastasis). Multivariate logistic regression analyses revealed that loss of membranous E-cadherin expression was the most significant phenotype associated with malignancy. Multivariate analysis showed that concomitant loss of membranous E-cadherin and increased cytoplasmic ALCAM expressions was the most significant adverse prognosticator in OSCC patients. Our study underscores the clinical relevance of aberrant expression of cell adhesion molecules in oral cancer. This is particularly important because differentiation is often a consequence of reduced or altered intercellular adhesion and maintenance of a differentiated phenotype of epithelial cells is critically dependent on the presence of functional adhesion complexes. However, the exact mechanism underlying these changes in oral carcinogenesis remains to be unraveled. In this context, siRNA mediated ALCAM knock down demonstrated increased cytoplasmic accumulation of β-catenin and E-cadherin, suggesting that there is an orchestrated modulation of these adhesion molecules which are dynamically controlled, supporting our clinical findings. The molecular mechanisms implicated are under investigation. We speculate that disturbances in this equilibrium might cause the cells to progress down the tumorigenic pathway. Notably, loss of E-cadherin and membranous β-catenin are hallmarks of epithelial- mesenchymal transition (EMT), defined as the loss of epithelial differentiation and gain of mesenchymal phenotype, that is associated with acquisition of aggressive traits by cancer cells, invasion and metastasis [44]–[46]. EMT is characterized by the loss of epithelial features, including reduced intercellular adhesion, a loss of epithelial cell polarity and an increase in cell motility. The presence of EMT has been suggested to be a likely predictor of OSCC progression and prognosis [47]–[50]. Aberrant activation of the Wnt signalling pathway is thought to initiate EMT in OSCCs, promoting tumor invasion and metastasis [51]–[54]. Alterations in expressions of Wnt pathway components, E-cadherin, β-catenin, vimentin, APC, Axin and GSK3 have been shown in OSCCs [47]–[55]. However, most EMT studies have been conducted in experimental systems and there is dearth of clinical data supporting the occurrence of EMT. Our study provides clinical evidence in support of EMT in OSCC.

In conclusion, our study underscores the clinical significance of loss of E-cadherin and β-catenin membrane expression in relation to ALCAM expression in early precancerous stage (dysplasia), their sustained deregulation in OSCCs and correlation with aggressive tumor behavior and poor prognosis, underscoring their potential as candidate biomarkers for disease prognosis. Further, our *in-vitro* studies support the clinical findings and suggest these dynamic changes in the cells adhesion system are likely to play pivotal roles in oral tumorigenesis.
